# Supplementary material for: Uncovering the Nutritive Profiles of Adult Male Chinese Mitten Crab (E. sinensis) Harvested from the Pond and Natural Water Area of Qin Lake Based on Metabolomics
Source: Foods. 2023 May 29;12(11):2178. doi: 10.3390/foods12112178 (PMC10253205; doi:10.3390/foods12112178)
Supplement: Supplementary file 1 [file foods-12-02178-s001.zip › foods-2368885-supplementary.pdf]

Supplementary Materials

Uncovering the nutritive profiles of adult male Chinese mitten crab (*E. sinensis*) harvested from the pond and natural water area of Qin Lake based on metabolomics

Yuhui Ye[1], Yulong Wang[1], Pengyan Liu[1], Jian Chen[1], Cunzheng Zhang[1*]

[1]Jiangsu Key Laboratory for Food Quality and Safety-State Key Laboratory Cultivation Base of Ministry of Science and Technology, Institute of Food Safety and Nutrition, Jiangsu Academy of Agricultural Sciences, Nanjing 210014, China

Figure legends

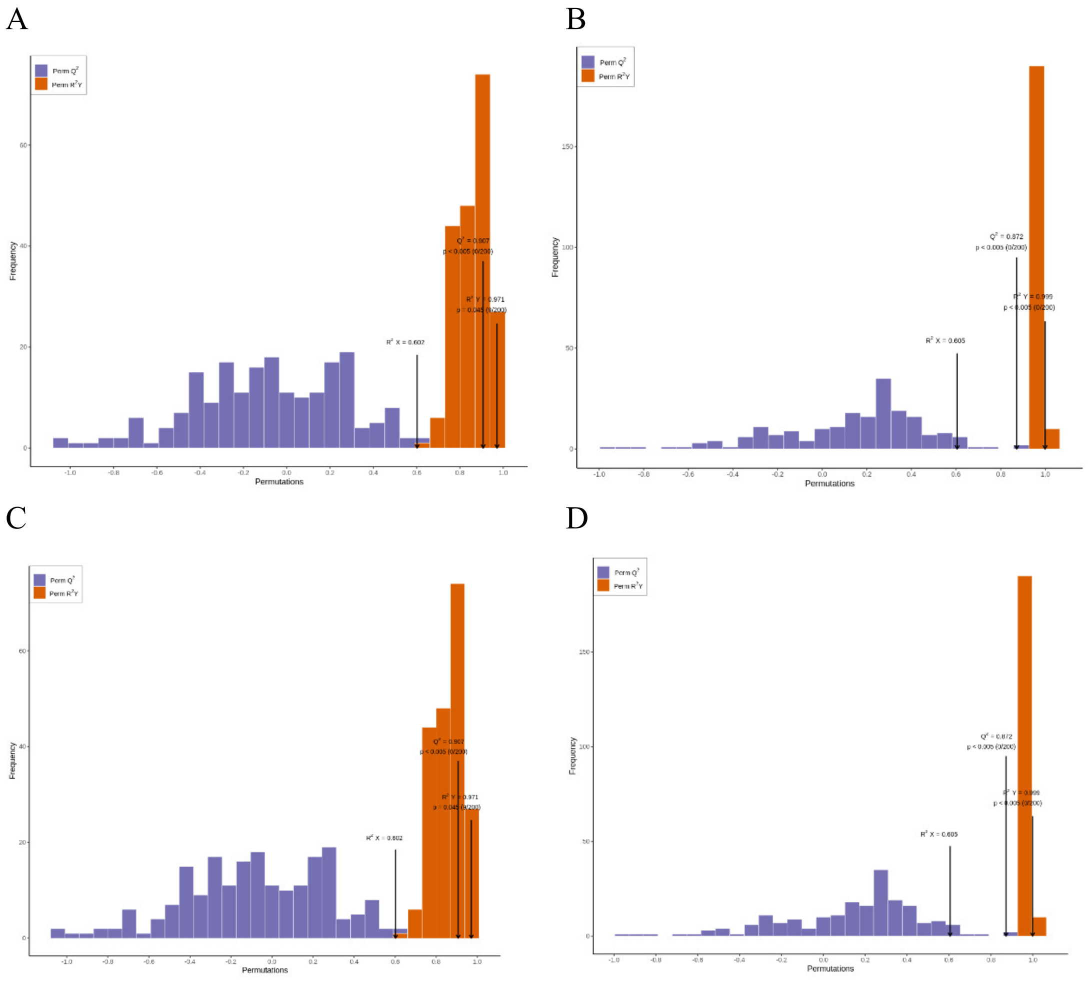

Figure S1. The OPLS-Permutation plot of the comparisons between N-M10 and P-M10 (**A**); N-M11 and P-M11 (**B**); N-M10 and N-M11 (**C**); P-M10 and P-M11 (**D**)

Table S1 The proximate composition of the meat and cream of *E. sinensis*

		**Meat**		
	**N-M10**	**P-M10**	**N-M11**	**P-M11**
**Water content (%)**	79.96 ±1.49^{a}	80.18±1.19^{a}	80.47±1.95^{a}	79.05±1.65^{a}
**Protein content (%)**	15.31±0.93^{b}	17.74±1.12^{ab}	17.45±1.33^{ab}	18.68±0.99^{a}
**Fat content (%)**	2.67±1.19^{b}	4.37±0.53^{a}	5.40±0.43^{a}	4.64±0.30^{a}
**Ash content (%)**	2.12±0.44^{b}	1.71±0.47^{b}	1.49±0.50^{b}	2.86±0.33^{a}
		**Cream**		
**Water content (%)**	57.19±1.32^{B}	63.14±1.38^{A}	62.16±0.92^{A}	65.30±1.81^{A}
**Protein content (%)**	8.11±0.84^{B}	9.70±1.26^{A}	9.60±1.27^{A}	10.65±1.01^{A}
**Fat content (%)**	38.85±1.19^{A}	38.85±1.28^{A}	28.52±1.13^{B}	38.19±0.43^{A}
**Ash content (%)**	1.37±0.31^{B}	1.97±0.35^{A}	1.37±0.30^{B}	1.48±0.24^{B}

Letters with the lower case mark the significant difference of each line of the meat samples (P<0.05); Letters with the upper case mark the significant difference of each line of the cream samples (P<0.05).
